# Spatio-temporal superconducting dynamics driven by THz fields from topological spintronic terahertz emitters

**DOI:** 10.1038/s41598-022-16377-y

**Published:** 2022-09-16

**Authors:** Björn Niedzielski, Dominik Schulz, Jamal Berakdar

**Affiliations:** Institut für Physik, Martin-Luther-Universität Halle-Wittenberg, 06099 Halle/Saale, Germany

**Keywords:** Mathematics and computing, Nanoscience and technology, Optics and photonics, Physics

## Abstract

Metastructures of spintronic THz emitters can be engineered to have a well-defined topology characterized by a topological charge. The emitted THz radiation possesses a phase-locked transversal and longitudinal components with the ratio of which being tunable by the topological charge of the underlying metastructure. The THz fields so produced are employed to drive and spatio-temporally modulate the superconducting order parameter in a type II superconductor. Using a time-dependent Landau-Ginzburg approach, it is demonstrated how the topology of the THz fields is reflected in a texturing of the superconducting phase and density. Full numerical simulations illustrate the emergence and the nanoscale steering of Abrikosov vortices as well as the local modification of the superconducting density and transport properties of nanoscale samples with different geometries. The study highlights the potential of metamaterials based on spintronic THz emitters as a coherent source for spatially and vectorially modulated THz radiation.

## Introduction

Electromagnetic waves allow the investigation of the electronic structure and resonant electronic transitions of superconductors (SC) in the UV frequency regime. In the (mid)infrared frequency range they are useful to assess for the roles of selective phonon modes excitation and the transient dynamics^1–5^. At lower frequencies (in the THz and below), SCs support a variety of sub-gap collective modes^6–8^ such as amplitude (Higgs) mode (massive excitations of the SC order parameter $\Psi ({\mathbf {r}},t)$ along the radial direction of the Mexican hat type free energy potential), acoustic phase modes, Bardasis-Schrieffer modes (fluctuations of subdominant order parameters), and others. In recent years, number of studies on these modes have been carried out using electromagnetic THz pulses (cf. Refs.^9–14^ and further references therein), and also the microwave regime has been addressed^15,16^. Generally, the coupling to far-zone transverse electromagnetic waves is weak, and a spatially-resolved study of the transient SC dynamics at the nanometer scale is hampered by the fact that THz (far) fields are basically spatially homogeneous on the nano to micrometer scale. Thus, it is desirable to find ways to generate THz fields with spatial and vectorial textures well below the (optical) diffraction limit with the aim to access for the THz linear and nonlinear response of SC materials to transverse and longitudinal fields. Recent approaches utilize near-field optics^14^.

Here, we propose and use spintronic THz emitters (STEs) in a geometry that allows generating propagating THz waves with a well-defined topology and having a vectorial texture on the sub-wavelength scale. The topology of the STEs results in THz fields having longitudinal and transversal components with a fixed phase relation. As a demonstration, the fields are applied to a type II SC and the spatio-temporal dynamics of $\Psi ({\mathbf {r}},t)$ in a micrometer scale sample are studied.

**Figure 1:**
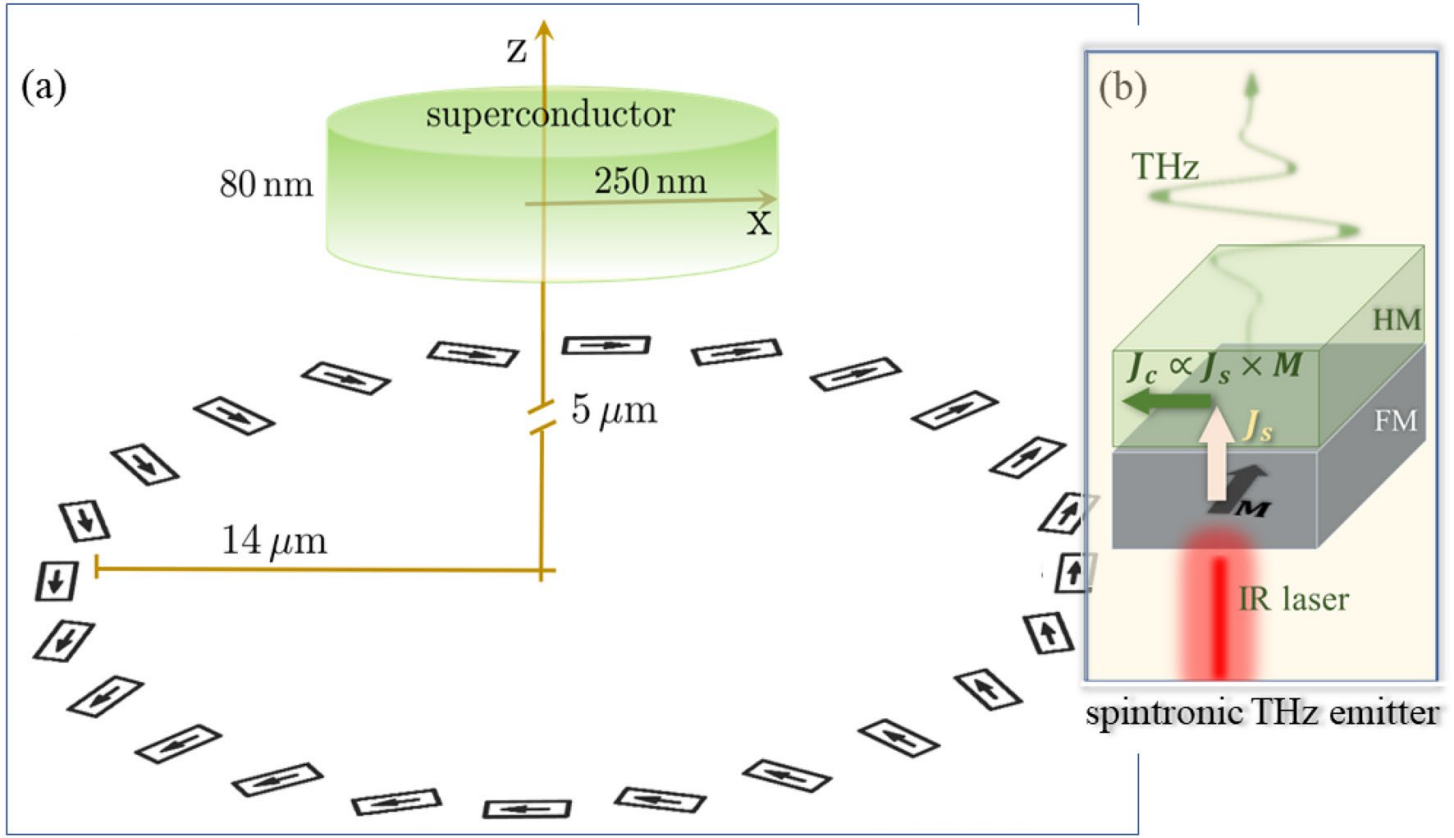
Setup of the considered system (not to scale). (**a**) A type II superconducting disc of 80 nm height and 250 nm radius is placed $5\, \upmu$m above the center of $\approx 28\, \upmu$m size metastructure of spintronic terahertz emitters (STEs). (**b**) An enlarged view of the key elements of one STE consisting of a ferromagnetic (FM) layer with magnetization ${{\textbf {M}}}$, interfaced with a heavy metal (HM) that acts as a strong spin orbit scatterer. A spin polarized current density $\mathbf {J_s}$ diffuses into the HM upon irradiation of the STE with an IR pulse. The inverse spin Hall charge current density pulse $\mathbf {J_c}\propto \mathbf {J_s}\times {{\textbf {M}}}$ results in THz emission. The arrows on each of the STEs in the metastructure indicate the local magnetization ${{\textbf {M}}}({{\textbf {r}}})$. $\mathbf {J_c}({{\textbf {r}}})$ is basically homogeneous in each one of the STEs and points perpendicular to ${{\textbf {M}}}({{\textbf {r}}})$ and parallel to the plane of the FM layer.

Experimentally, there have been remarkable advances in the generation of THz radiation exploiting spin-dependent transport in layered spintronic elements (hence, the name STEs)^17–23^. The basic unit (illustrated in Fig.1b) of the STE is a metallic ferromagnet (FM) interfaced with a heavy normal metal that acts as a strong spin orbit scatterer (for more details, we refer to Refs. ^24–28^). A nonequilibrium electronic population triggered in the ferromagnet by irradiation with an infrared laser is accompanied with a spin polarized current $\mathbf {J_s}$ that diffuses in the heavy metal. Due to the inverse spin Hall effect, a decaying charge current density $\mathbf {J_c}\propto {\mathbf {M}}\times {\mathbf {J}}_s$ is generated leading to the THz emission (${\mathbf {M}}$ is the magnetization direction of the FM). Metastructures of STEs^23^ (such as those shown in Fig. 1) render possible a sub-wavelength spatial texturing of the polarization properties of the propagating THz field. Here, we discuss how to design the metastructure as to endow the fields with a well-defined topology resulting in phase-locked transversal and longitudinal components of the emitted field. The calculated THz pulses are used as input to drive the SC order parameter in a sample placed at a micrometer scale distance away from the THz generating metastructure (Fig. 1a). The utility of calculated THz fields for imprinting nanoscale topological features (such as Abrikosov vortices) on a type II SC is demonstrated. In particular, as shown below, the vortices can be created and pinned by the applied field. Vortices play a central role in the behavior of SC^29^. For example, when driven by an electric current, they cause resistive voltage and energy dissipation in the SC. Also, steering vortices might be useful for other applications such as topological quantum computing^30–32^. The THz pulse can thus be used to spatio-temporally control this contribution to the electrical resistance. Beyond the presented simulations, the THz fields emitted by the STEs metastructures offer further opportunities to explore aspects of SCs, in particular, the non-local and nonlinear longitudinal and/or transverse response.

## Metastructure of STEs

In this study, we consider STEs that are individually rotated in a way that the global magnetization of the STEs resembles a quadrupole-like texture (cf. Fig.1a). The discreteness of the STEs (their size and shape) is essential in that it ensures the stability of the local magnetization ${{\textbf {M}}}$ and hence the direction of the current density ${{\textbf {J}}}_c({{\textbf {r}}})$ leading to THz emission (cf. Fig.1b). In this way, the polarization of the emitted field is controllable by the local magnetization of the STEs and can be textured on the nanometer scale. For a possible experimental realization, one may use an external quadrupolar field^22^ created by two magnets with opposing magnetizations. After the initial state is formed, the external field may be switched off. Another possibility relies on the use of two magnetic materials with different stiffness, where two opposing magnetic fields are applied. First, a strong field switches the magnetization of all STEs. Then, a second weaker field is applied, switching the magnetization of the STEs with the softer FM layers. The following relaxation forms the desired pattern. Higher multipole orders of the magnetization structures are possible as well. For our study we consider the case where the emitters are designed to have a particular topology characterized by the topological number $q=\frac{1}{2\pi }\int _0^{2\pi }\left[ \partial _{\varphi }{\mathbf {m}}\times {\mathbf {m}} \right] _z \,\mathrm {d}\varphi$, where ${\mathbf {m}} = \frac{{\mathbf {M}}}{M}$ is the unit magnetization direction and $\varphi$ is the azimuthal angle. Changing the number of twists of ${\mathbf {m}}$ in $2\pi$, the topological charge *q* can be changed.

For a description within a continuous framework, one may assume a space-filling limit of the STEs (forming an annulus). Then, the axial symmetry is exploited for two setups consisting of different azimuthal mode numbers $m=2$ and $m=-2$. The emitted electric field at the surface of the annulus is described by $\mathbf {{E}}_0 = E_0\left[ \cos ((m-1)\varphi )\,\hat{\varvec{\mathrm {e}}}_x - \sin ((m-1)\varphi ) \,\hat{\varvec{\mathrm {e}}}_y\right]$. Here, $E_0$ is the amplitude of the electric field, $m-1$ is equal to the topological charge *q*, and $\,\hat{\varvec{\mathrm {e}}}_{\alpha }$ are the unit vectors along $\alpha$ (more details about the simulations are in “Methods” section). For the case of $q=0$, the topology of the STEs becomes trivial^23^, for example, in this case the STEs magnetizations in Fig.1a point all such that ${\mathbf {m}}({\mathbf {r}})\parallel \hat{{\mathbf {e}}}_\varphi$ (azimuthal structure) or radially ${\mathbf {m}}({\mathbf {r}})\parallel \hat{{\mathbf {e}}}_r$ (radial structure). The emitted THz fields are then radially or azimuthally polarized. The latter, for instance, has only a transverse component^23^. Hence, imprinting a topology onto the STEs allows the controlled coherent buildup of transverse and longitudinal components of the emitted radiation (the field components and the resulting SC dynamics are discussed below, e.g., in Fig. 4a,b).

Typically, the THz radiation from STEs is broadband. Here, we employ an almost monochromatic field to study the dynamics of SC (the used envelope of the pulse in frequency space is narrow), as given and explained in details in the Method section. There, we also discussed a self-similar metastructure for producing THz fields leading to the same topology but with an enhanced field strength.

In view of a pump-probe experiment or/and to avoid heating effects of the SC sample, one may use a IR pulse with a donuts shape intensity profile (e.g., a Bessel beam)^33^ or a combination of a Bessel beam (acting on the STEs to probe and pump the SC) and a Gaussian beam (pumping or probing the SC), similar to recent studies^34,35^ utilizing this type of pulse combination.

## Setup and results

As illustrated by Fig. 1a, a SC cylinder of $80\,$nm thickness and $250\,$nm radius is placed $5\,\upmu$m away from the center of the STEs structure. The overall size of the STEs metastructure is on the micrometer scale. Nonetheless, the vector potential ${\mathbf {A}}_T$ of the emitted THz field at the position of the SC is spatially (orbital part of ${\mathbf {A}}_T$) and vectorially (polarization or spin part of ${\mathbf {A}}_T$) textured on the nanometer scale (cf. “Methods” section and below). Hence, we expect modifications of the superconducting phase and density; an expectation that we will quantify below with full numerical simulations.

We use cylindrical coordinates ${\mathbf {A}}_T({\mathbf {r}})=\mathbf{A}_T(\rho ,z, t)$ and a time envelope with central frequency in THz (e.g., $\omega = 10\,$THz). At first we perform electromagnetic/micromagnetic simulations to obtain ${\mathbf {A}}_T$ for the given metastructure of STEs. Then, we insert the obtained vector potential (together with any other external magnetic fields with corresponding vector potential ${\mathbf {A}}_{\mathrm {e}}$) into the time dependent Ginzburg-Landau equations^36^

1 $$\begin{aligned}&\gamma \left( \hbar \partial _t \Psi + \mathrm {i} q_{\mathrm {s}} \phi \Psi \right) + \Psi \left( \beta |\Psi |^2 - |\alpha | \right) + \frac{1}{2m_{\mathrm {s}}}\left( \frac{\hbar }{\mathrm {i}}\nabla - q_{\mathrm {s}} {\mathbf {A}} \right) ^2 \Psi = 0, \end{aligned}$$

2 $$\begin{aligned}&\frac{1}{\mu _0}\nabla \times \nabla \times {\mathbf {A}}_{\mathrm {s}} = {\mathbf {j}}_{\mathrm {s}} -\sigma \left( \partial _t{\mathbf {A}}_T + \partial _t{\mathbf {A}}_{\mathrm {s}} + \nabla \phi \right) , \end{aligned}$$

3 $$\begin{aligned}&{\mathbf {j}}_{\mathrm {s}} = \frac{\mathrm {i} q_{\mathrm {s}} \hbar }{2m_{\mathrm {s}}}\left( \Psi \nabla \Psi ^{\star } - \Psi ^{\star } \nabla \Psi \right) - \frac{q_{\mathrm {s}}^2}{m_{\mathrm {s}}}\left| \Psi \right| ^2{\mathbf {A}} . \end{aligned}$$

${\mathbf {A}}_{\mathrm {s}}$ is the induced vector potential in the SC, and ${\mathbf {A}} = {\mathbf {A}}_{\mathrm {s}} + {\mathbf {A}}_T + \mathbf{A}_{\mathrm {e}}$. The Landau coefficients $\gamma$,$\alpha$ and $\beta$ are temperature dependent material parameters. $n_{\mathrm {s}} = |\Psi |^2$ is the local density of Cooper pairs. $n_{\mathrm {s}} = 1$ indicates perfect superconductivity and $n_{\mathrm {s}} =0$ is normal conductance. $m_{\mathrm {s}}$ and $q_{\mathrm {s}}$ are the mass and charge of a Cooper pair. Writing $\Psi = \Psi _0 \mathrm {e}^{\mathrm {i}\theta _{\mathrm {s}}}$ exposes the relation between the supercurrent density Eq. (3) and SC phase gradient

4 $$\begin{aligned} {\mathbf {j}}_{\mathrm {s}} = \frac{q_{\mathrm {s}}}{m_{\mathrm {s}}} \Psi _0^2\left( \hbar \nabla \theta _{\mathrm {s}} - q_{\mathrm {s}} {\mathbf {A}} \right) . \end{aligned}$$

As an example, the numerical simulations are performed for Nb at a temperature of $T = 4\,$K^37^. The scalar potential $\phi$ appearing in Eqs. (1) and (2) is eliminated by choosing the gauge $\mu _0 \sigma \phi = - \nabla \cdot {\mathbf {A}}$^38^ (here, $\sigma$ is the normal state conductivity). This gauge choice imposes the following boundary conditions on the vector potential

5 $$\begin{aligned} {\mathbf {A}} \cdot {\mathbf {n}} = 0. \end{aligned}$$

where ${\mathbf {n}}$ is the outward unit vector normal to the superconducting domain. For conciseness, no further external magnetic fields are applied (${\mathbf {A}}_{\mathrm {e}} = 0$), and hence

6 $$\begin{aligned} {\mathbf {A}}_{\mathrm {s}} \cdot {\mathbf {n}} = - {\mathbf {A}}_T \cdot \mathbf{n}. \end{aligned}$$

The boundary condition of Eq. (1) is

7 $$\begin{aligned} \left( \frac{\hbar }{\mathrm {i}}\nabla \Psi - q_{\mathrm {s}} \Psi {\mathbf {A}} \right) \cdot {\mathbf {n}} = 0 \end{aligned}$$

which enforces ${\mathbf {j}}_{\mathrm {s}} \cdot {\mathbf {n}} = 0$. For Eq. (2) we set the following boundary condition

8 $$\begin{aligned} {\mathbf {B}}_{\mathrm {s}} \times {\mathbf {n}} = \left( \nabla \times {\mathbf {A}}_{\mathrm {s}} \right) \times {\mathbf {n}} = 0. \end{aligned}$$

Equation (8) is a consequence of the commonly used boundary condition^38–40^

$$\begin{aligned} \left( {\mathbf {B}}_{ext} - \nabla \times {\mathbf {A}}\right) \times {\mathbf {n}} = 0, \end{aligned}$$

which is applied when the second TDGL equation is solved for the complete vector potential ${\mathbf {A}} = \mathbf{A}_{ext}+{\mathbf {A}}_s$. In our case, the external vector potential was calculated as input for the TDGL-simulations and we assumed that the presence of the superconductor does not affect the STE-emitters (due to the relatively large distance between them). In this case, the boundary condition for the vector potential ${\mathbf {A}}_s$ admits the form of (8). In a more accurate scenario, this boundary condition should be applicable at large distances from SC. Implementation of open boundary conditions is, however, limited by computational resources. Therefore, Eq. (8) is commonly used, which allows taking (to some extent) into account the self-interaction of the superconductor with its stray fields.

Details of the numerical implementations of the SC dynamics in the simulated THz fields are in the “Methods” section.

Eddy currents appearing in Eq. (2) are associated with gradual heating of the sample. However, for simplicity, we assume the temperature to be constant in all calculations.

## Discussions

**Figure 2:**
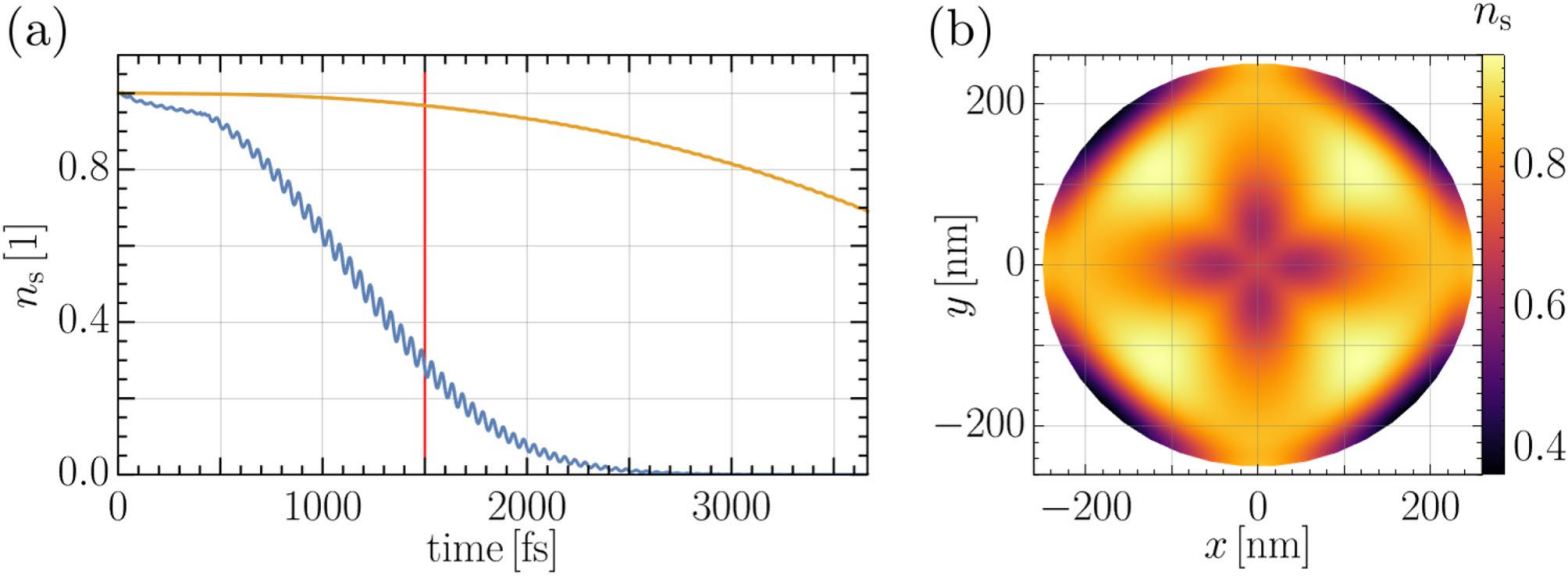
(**a**) The minimum (blue curve) and maximum value (orange curve) of the local density of Cooper pairs $n_s$ under the influence of the vector potential. (**b**) Snapshot of $n_s$ at $z=0$ and $1500\,$fs indicated in (**a**) by a red line.

**Figure 3:**
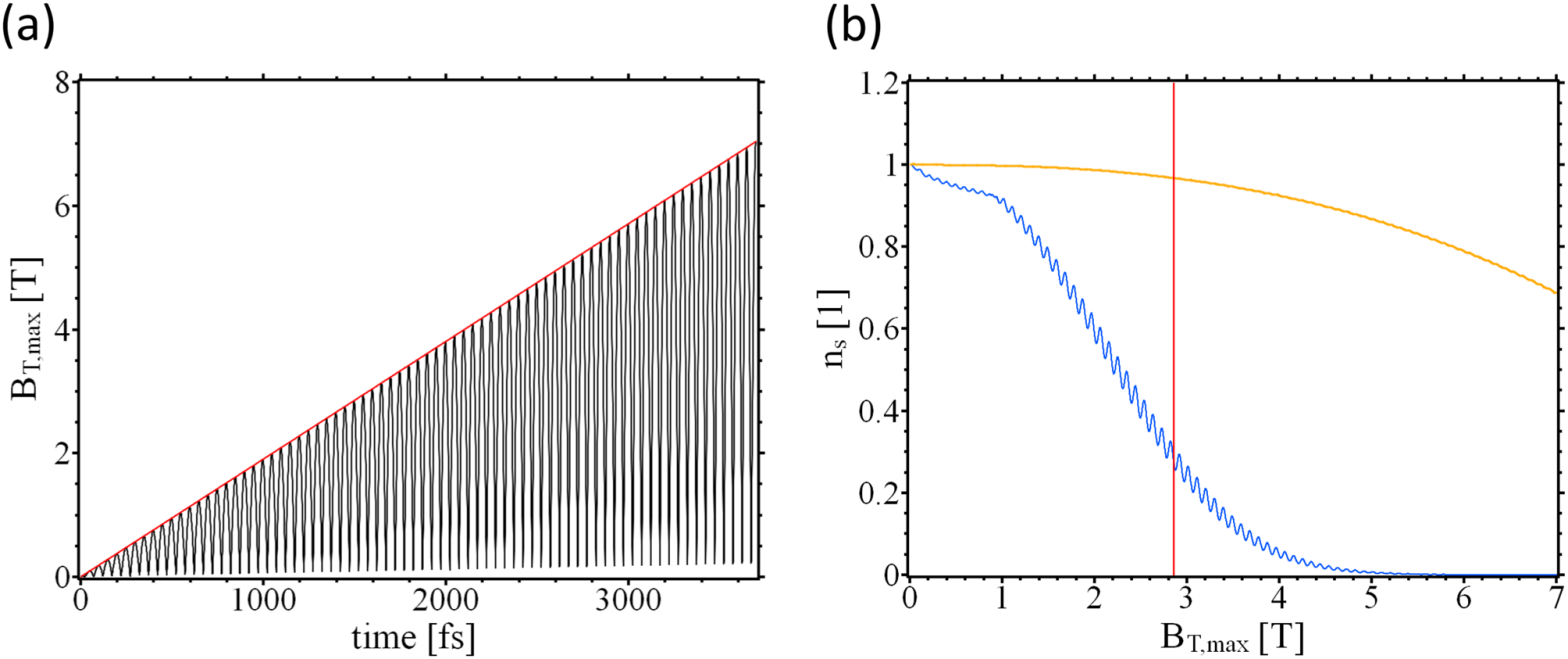
(**a**) Local maximum value of $B_\mathrm {T,max}$ (black curve) and its envelope (red curve) in the sample over time. (**b**) The minimum (blue curve) and the maximum value (orange curve) of the local density of Cooper pairs $n_s$ over the envolop of $B_\mathrm {T,max}$ displayed in (**a**).

We write for the THz field vector potential ${\mathbf {A}}_T=A_{T0}(t){\mathbf {A}}_{T1}$ with, ${\mathbf {A}}_{T1}({\mathbf {r}},t)= {\mathbf {A}}_{T1}({\mathbf {r}},t+T)$ and $T=2\pi /\omega$. To follow the evolution of the SC $A_{T0}$ is ramped up at the rate $a_T = 16.59 \text {T}\mu$m/ps until a maximum value $A_{T0}=59.733 \,\text {T}\mu$m is reached. In the “Methods” section an analysis is performed of the dominant frequencies contained in the ramped THz field. Of interest are the maxima and minima of $n_{\mathrm {s}}$ which are displayed in Fig. 2a. The order parameter amplitude oscillates as the field develops in time. For sufficiently strong fields superconductivity can be suppressed. The SC state does not break down homogeneously in the entire sample but respects the local distribution of currents, as set by the vector potential and subject to the boundary conditions (cf. Eq. (4)). This can be seen in Fig. 2b. It should be mentioned that we used relatively strong fields in our calculations. Currently, the reported local maximum amplitude of the B-field are in the range of $B_\mathrm {T,max}\approx 0.1 T$. The maximum field values in our calculation were on the order of Tesla (cf. Fig. 3).

**Figure 4:**
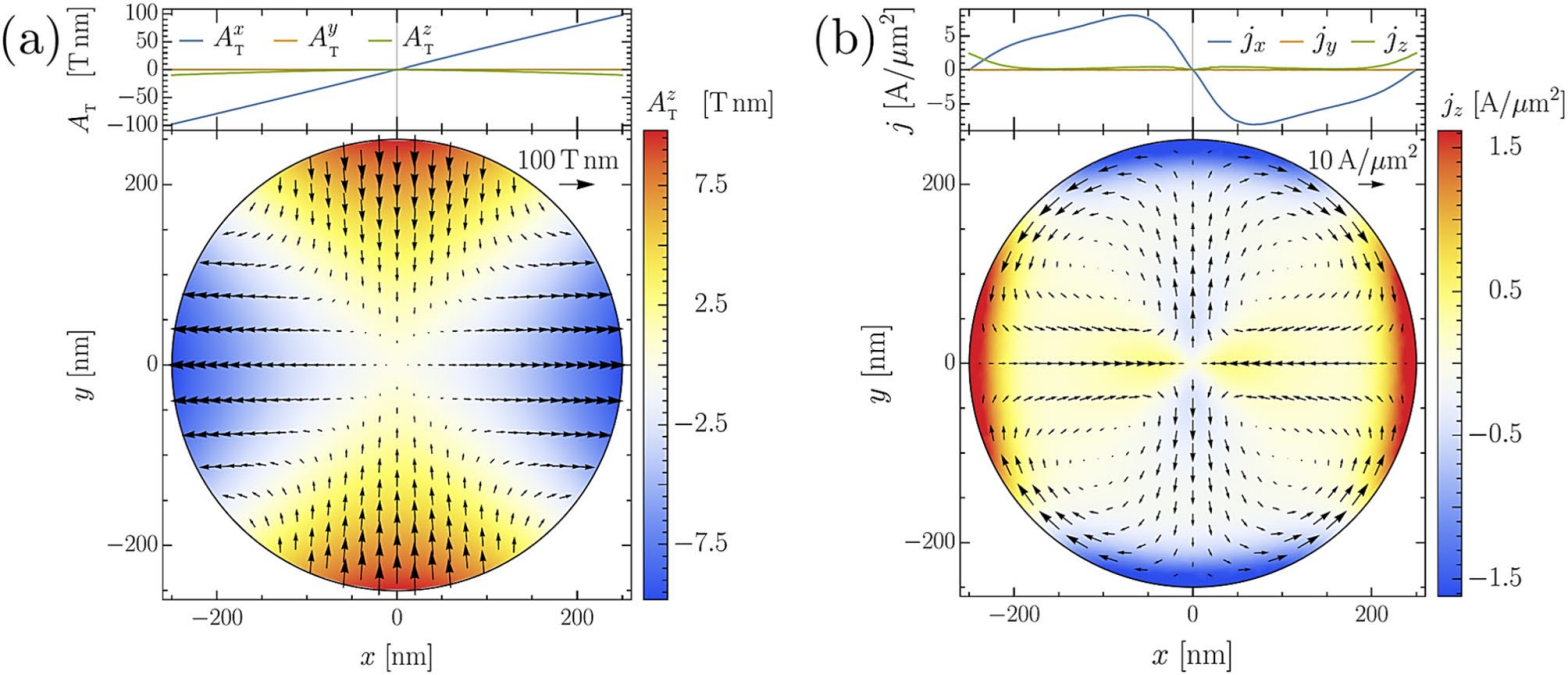
(**a**) The calculated vector potential for the STEs metastructure shown in Fig. 1 at the position of the SC sample. (**b**) The total current density (**b**) in the superconductor in the process of ramping up the fields at $z=0$ and $t=0.5$ ps (upper insets show the dependence in the *x* direction at $y=0=z$).

We note that $\Psi$ is mostly affected by the eddy currents since their magnitude is several orders higher than that of $\mathbf{j}_{\mathrm {s}}$. For very high field strengths the normal currents start to destroy superconductivity at the center of the SC. Also the currents flowing around the edges become relevant and lead to the formation of several superconducting islands in the sample. The breakdown of superconductivity at the center of the sample is not only a consequence of the strong current flow. Figure 4a evidences that the vector potential ${\mathbf {A}}_T$ changes its spatial orientation rapidly at the disc center. e.g., the *x* component changes from positive to negative on a length scale that is comparable to the GL coherence length. The superconducting phase field tends to follow the external potential. The steep phase gradient created in this way also affects the order parameter density and leads to a gradual suppression of the superconductivity at the disc center. In turn, the reduction of $\Psi$ affects the supercurrents since their magnitude is directly proportional to $n_{\mathrm {s}}$. In contrast, the normal current distribution is only slightly affected by the breakdown of superconductivity. The distribution of the total current is shown in Fig. 4b. The overall features of the current align with its relation Eq. (4) to the vector potential (Fig. 4a) and the imposed boundary conditions forcing the current density to flow around the cylinder which leads to the formation of local whirls in the current density.

One may expect the current flow to be governed by the spatial variation of the applied vector potentials. The time evolution of the phase field $\theta$, and the order parameter amplitude $n_\mathrm {s}$ show, however, a non-trivial time evolution. Technically, this is because both quantities are calculated from coupled and nonlinear partial differential equations. On the other hand, the dynamics of $\Psi$ happen on a finite time scale and the order parameter can not adapt instantaneously to the external vector potential. These effects alter the current flow in the disc leading to a quite involved time evolution.

**Figure 5:**
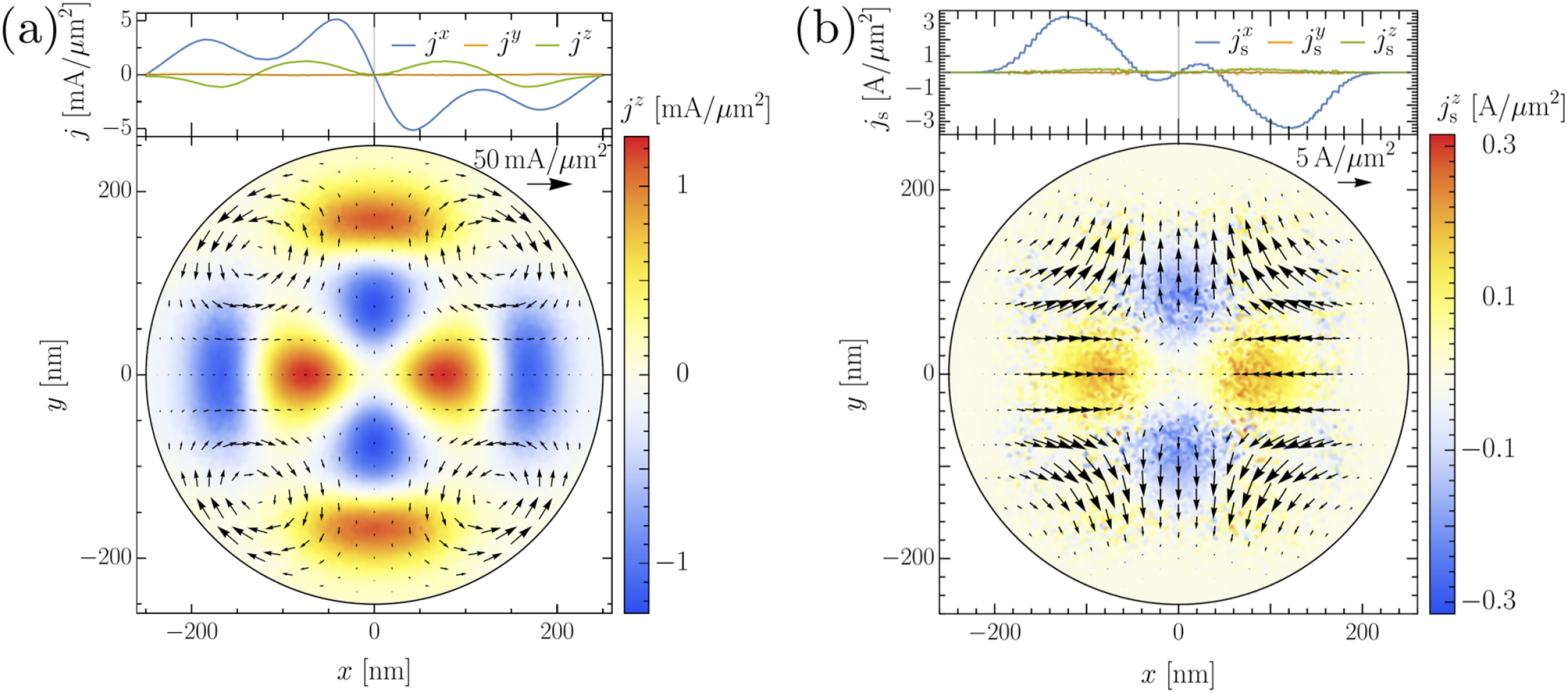
Spatial distribution in the *xy* plane and at $z=0,\ t=1.5$ ps of the total current density (**a**), and of the supercurrent (**b**) in the superconductor while the THz field fades away. Upper insets show the corresponding values along the *x* direction for $y=0$.

To study the SC behavior during switching off the field, we monitor the SC dynamic starting from the initial value $A_{T0}=59.733\,$Tμm and decreasing it to zero (after $t=0.18\,$ps) at a rate of $a_{T} = 331.8\,$Tμm/ps. The order parameter is strongly suppressed when the field is switched off. The field was ramped down with a rate that is much higher than the initial ramping rate. Therefore, the normal currents generated by the time variation of ${\mathbf {A}}$ are higher in this case and reduce the superconducting order parameter. In this process the phase $\theta _{\mathrm {s}}$ develops a steep gradient which leads to delicate supercurrent distributions (see Fig. 5a,b), which is mostly in-plane. The time evolution of the phase suggests that also vortex-antivortex (VAV) pairs are created in the rapid decrease of $|\Psi |^2$ (not shown here). They appear as little knots in the phase field close to the border of the sample. But since no additional magnetic field is applied the VAV-pairs are highly unstable and annihilate eventually. For the stabilization, a static magnetic field is needed which is by itself not capable of creating the VAV pairs but it preserves them, once created by ${\mathbf {A}}_T$. The normal current also displays a more involved time evolution than in the previous case. A number of magnetic whirls along the sample edges are generated. However, these whirls are not equivalent to the conventional superconducting vortices since there is no phase singularity and no normal conducting vortex core.

**Figure 6:**
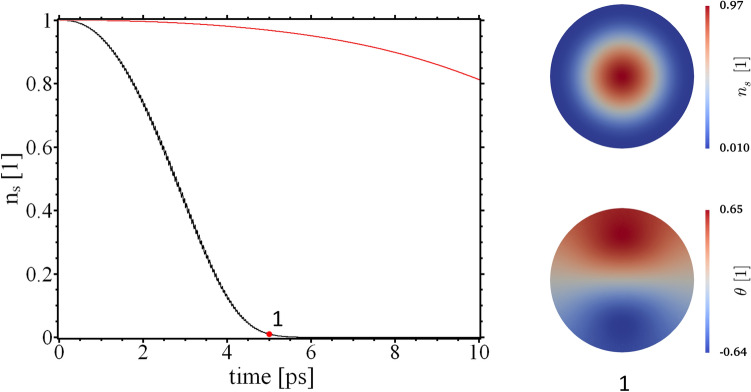
Left: The minimum (black curve) and maximum value (red curve) of the local density of Cooper pairs $n_\mathrm {s}$ under the influence of the plane-wave vector potential. Right: Snapshot of $n_\mathrm {s}$ and $\theta$ at $z=100\,$nm and $100\,$ps indicated by a red dot in the left plot.

To illustrate the role of the orbital and vectorial texture of ${\mathbf {A}}_T$, we investigate how the superconductor reacts to a plane-wave THz field (no spatial textures). This amounts to using the vector potential

9 $$\begin{aligned} {\mathbf {A}}_T(x,t) = -a_Tt\sin \left( \frac{2\pi }{\lambda }\left( ct-x \right) \right) \mathbf{e}_y+\nabla u \end{aligned}$$

as an input for the first time dependent GL-equation. The function $u({\mathbf {r}}, t)$ is chosen such that $\nabla \cdot \mathbf{A}_\mathrm {T} = 0$ and ${\mathbf {A}}_\mathrm {T}\cdot {\mathbf {n}}=0$ holds at all times. The corresponding magnetic field has only components in the *z* direction. To reduce the numerical efforts, the calculations are performed for a circular SC of radius $R = 250\,$nm located in the plane $z = 100\,$nm. Furthermore, we neglect the SC self field $\mathbf {\mathbf {A}}_\mathrm {s}$ which is allowed in the cases where $\lambda _\mathrm {eff}\gg \xi _\mathrm {GL}$^41^. As before, we choose the frequency of the potential to be $f=10\,$THz with a corresponding wavelength $\lambda = 30 \,$ $\mu$m, i.e $\lambda \gg R$. Thus, the space variation of $B_\mathrm {T} = \nabla \times {\mathbf {A}}_\mathrm {T}$ is negligible and the external field is basically homogeneous in space. The prefactor in Eq. (9) has the value $a_\mathrm {T} = 0.41\,$Tμm/ps, meaning that after $t=5\,$ps the external magnetic field has reached a value of $B_\mathrm {T}=0.39\,$T. The time evolution of the system is shown in Fig. 6. As in the previous calculations, the effect of $A_T$ on the superconductor consists of gradual suppression of the order parameter. However, for the plane-wave vector potential, the suppression of $\Psi$ happens solely around the sample edges, whereas the center remains superconducting, highlighting so the role of finite-size effects. With an increasing external field strength, the normal conducting ring domain grows until the material becomes completely normal conducting again. An interesting observation is that the phase of the order parameter is only slightly affected by the external field. In contrast, the field created by the spintronic THz emitters results in a strong modulation of the order parameter phase. Also, the normal conducting domains at intermediate field strengths have a more complicated structure.

**Figure 7:**
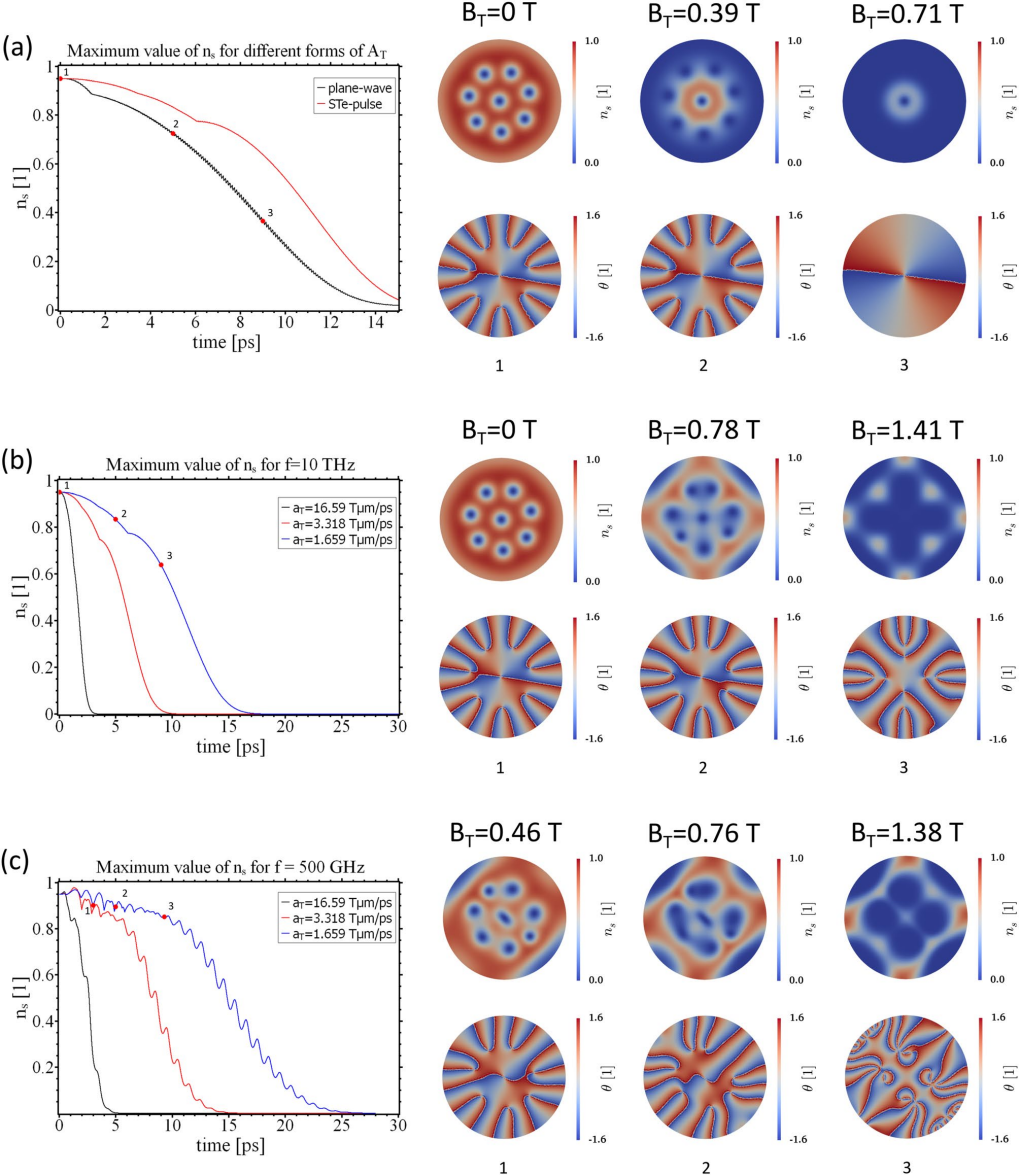
Maximum value of $n_\mathrm {s}$ for $B_\mathrm {e} = 0.17\,$T and different THz field pulses. (**a**) vortex state under application of a plane-wave pulse (black curve and $a_\mathrm {T} = 0.41\,$Tμm/ps) and a STE-pulse (red curve and $a_\mathrm {T} = 1.659\,$Tμm/ps) for $f=10\,$THz. (**b**, **c**) STE-pulse with $f=10\,$THz and $f=500\,$GHz and different ramping rates $a_T$ of the external vector potential. Panels on the right: order parameter amplitude $n_\mathrm {s}$, phase $\theta$ and maximum value of $B_\mathrm {T}$ for the points in time indicated on the left.

In light of the above observations, the question arises of how an ensemble of superconducting vortices reacts to structured THz field pulses. To address this issue, an additional external magnetic field ${\mathbf {B}}_\mathrm {e} = B_\mathrm {e} {\mathbf {e}}_\mathrm {z}$ is applied to the sample with $B_\mathrm {e} = 0.17\,$T. For such a field, the equilibrium state of the system consists of a circular arrangement of 8 vortices (cf. Fig. 7a)a. Due to finite-size effects, the vortices experience a strong confining potential which is brought up by the Meissner currents flowing around the sample edges. As a consequence, the vortex lattice reflects the symmetry of the sample. In the next step, we investigate how the state under finite $\mathbf{B}_\mathrm {e}$ is affected by THz field pulses of different topology, amplitude, and frequency. We start by comparing the vortex behavior under a conventional plane-wave THz field pulse with the vortex dynamics steered by the STEs described above. In Fig. 7a, one can see that the application of the plane-wave field leads to a stronger order parameter suppression compared to the STE-pulse, despite a weaker field-magnitude. This is because the fields lead to different current distributions in the sample, which in turn affect the order parameter differently. Another observation is made when comparing the right panels of Fig. 7a,b): The plane-wave pulse is able to drive the vortices out of the sample for sufficiently high field amplitude. In contrast, the STE-pulse preserves the topology of the phase field and the vortex lattice experiences a structural change without vortex creation or annihilation. It should be noted that the qualitative dynamic behavior of the vortex ensemble is found to be independent of the rate at which the external field is increased.

The frequency of $f=10\,$THz is substantially higher than the gap frequency of Nb $f_{Nb}=2\Delta _{\mathrm {Nb}}/h=701\,$GHz and the radiation field has, in addition, a pair breaking effect on the superconducting condensate. In this respect, cuprate superconductors with gaps on the order of $\Delta \sim 20\,$meV would be better-suited^42^. To avoid effects related to pair breaking, we investigated how the dynamics of the system change if the pulse-frequency is reduced down to $f=500\,$GHz (cf. Fig. 7c). The general behavior of $n_\mathrm {s}$ is the same as in the case of $f=10\,$THz. However, the maximum value of $n_s$ exhibits stronger oscillations since the order parameter is offered more time to adapt to the more slowly varying external field. The vortex lattice is also stronger affected by the pulse with $f=500\,$GHz. In particular, the vortex cores are notably deformed even at moderate field strength values. In addition, we find that the low-frequency pulse has a certain tendency to make the vortices clump together (see panel two in Fig. 7c). This tendency becomes even more clear in the high field regime, where we have essentially four giant vortices instead of 8 distinct vortex cores. Furthermore, the phase field $\theta$ indicates the temporal appearance of vortex-antivortex pairs. As in previous calculations, the observed vortex dynamics are essentially independent of the rate at which the external field increases, as long as the frequency is kept constant.

**Figure 8:**
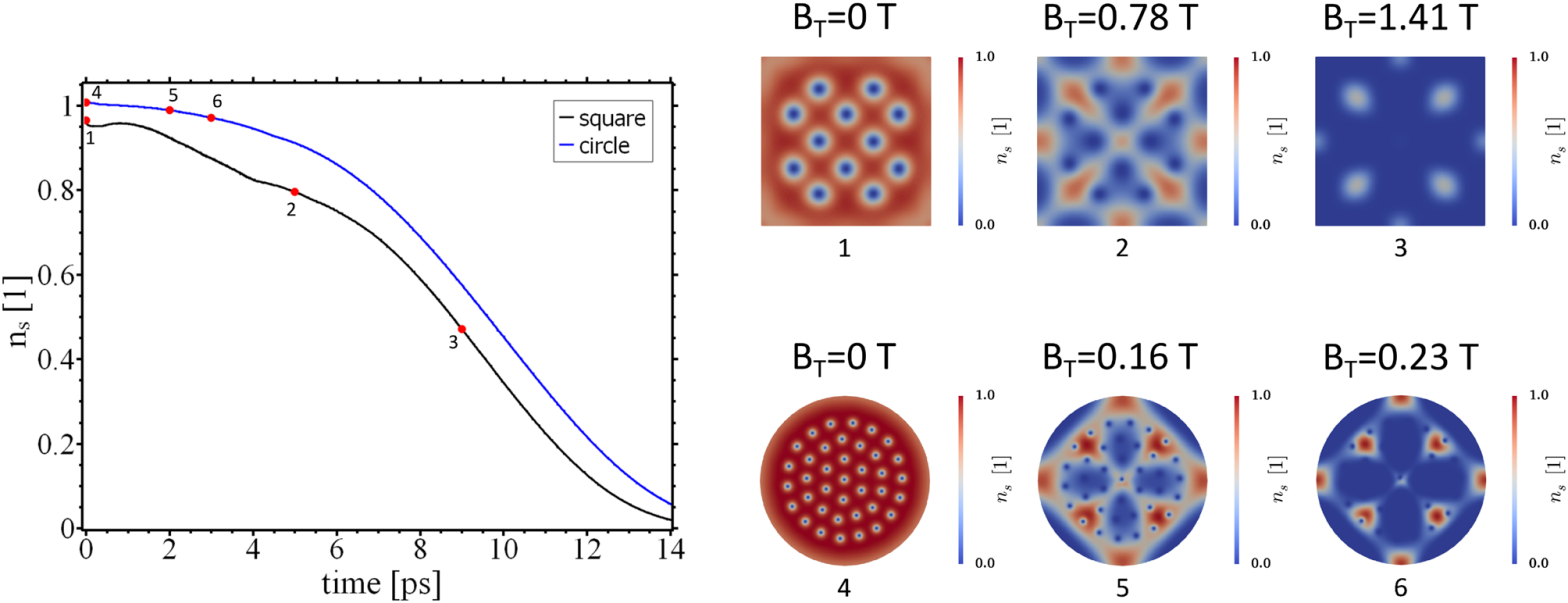
Left: maximum value of $n_\mathrm {s}$ for $B_\mathrm {e} = 0.17\,$T and application of a STE-pulse with $a_T = 1.659$T$\upmu$m/ps. The black curve corresponds to a square sample of side length $a=250\,$nm and the blue curve to a disc of radius $r=1\,\upmu$m. Panels on the right: order parameter amplitude $n_\mathrm {s}$ and maximum value of $B_\mathrm {T}$ for the points in time indicated on the left.

To highlight the role of geometric confinement in our system we performed the calculations discussed above on samples of different geometry and spatial extension. The applied STE-pulse has a frequency $f=10\,$THz and a ramping factor $a_T = 1.659$Tμm/ps. The sample is a square with side length $a = 250\,$nm or a superconducting disc of radius $r = 1\, \upmu$m (cf. Fig. 8).

A consequence of the modified geometry is inferred from the upper panels on the right, where the vortices form a simple cubic lattice in the square sample. The current flow around the edges of the square creates an energetic barrier that prevents an immediate relaxation of the vortex lattice into a hexagonal arrangement. However, if this vortex ensemble is subject to the THz pulse, one can see a very similar behavior as in the case of the THz field-driven disc with $r=250\,$nm. With increasing the amplitude of the magnetic field, the vortex cores grow in size until most of the sample becomes normal conducting. But also, in the square sample, the THz field pulse is not changing the topology of the order parameter phase-field $\theta$ and the vortex number is preserved (not shown here). The situation is different if we return to the superconducting disc with an increased radius of $r=1\, \upmu$m (cf. lower panels on the right in Fig. 8). This system is less affected by geometric confinement and the vortex lattice has adopted a hexagonal symmetry. An important difference from the previous systems is the higher sensitivity of the order parameter to the applied THz field. Already at a maximum value of $B_\mathrm {T}=0.16\,$T, one can observe the formation of four almost normal conducting domains. At $B_\mathrm {T}=0.23\,$T, the system is mostly normal conducting. Due to the presence of the fully developed vortex lattice, the larger system size and the lesser importance of the boundary currents, the total current distribution in the disc differs from that of the previous systems. That also means the currents induced by the THz field pulse have a different impact on the order parameter development. For even larger systems, the finite spatial extent of the radiation field will become an important factor as well, and superconductivity is only suppressed at the center of the disc. The normal conducting domains induced by the time-varying magnetic field and the local heating of the sample then might provide another way to detect and characterize the properties of the pulse. Particularly, the formation of stable vortex clusters of characteristic shape inside the normal domains might serve as a fingerprint for the specifics of the radiation field^43^.

**Figure 9:**
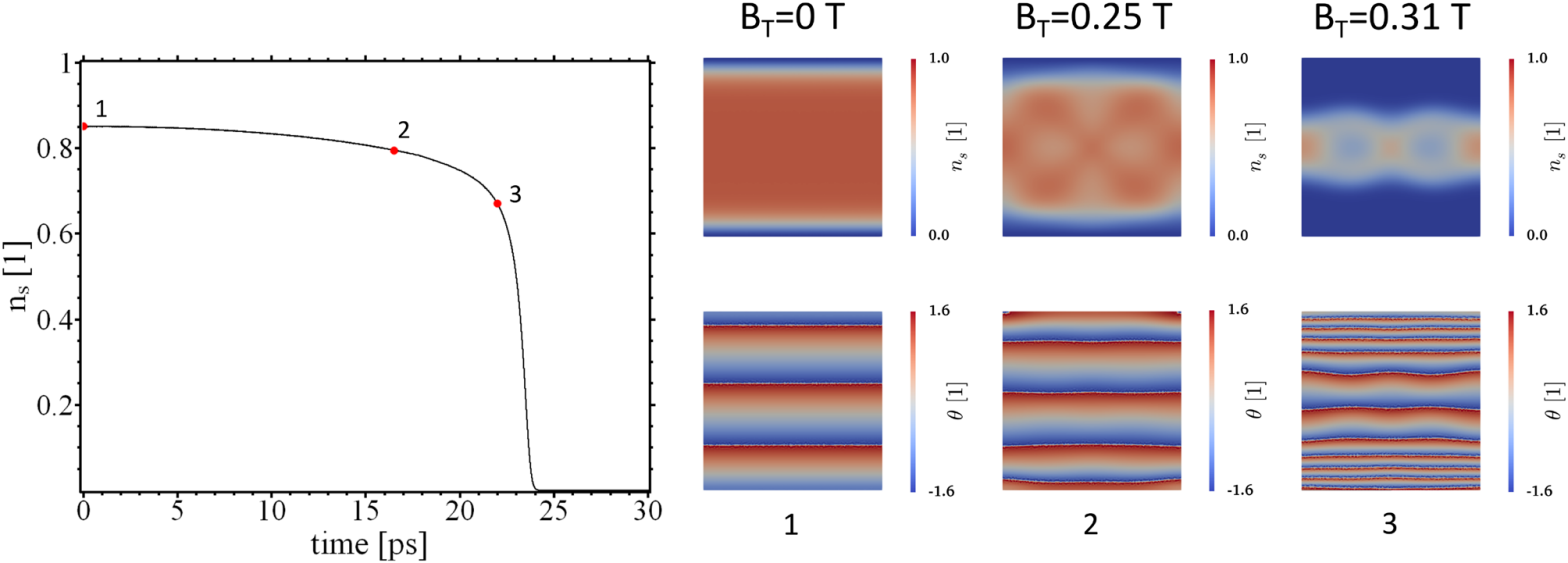
Left: maximum value of $n_\mathrm {s}$ for $j_\mathrm {e} = 0.95 j_c\,$ and application of a STE-pulse with $a_T = 0.166$Tμm/ps. Panels on the right: order parameter amplitude $n_\mathrm {s}$ and phase $\theta$ for the points in time indicated on the left.

To explore the effect of the THz fields on transport, we inject an electric transport current into the superconductor in the presence of the STE generated THz field pulse. The idea is to simulate a system which is similar to a superconducting single-photon detector. It is known that in such systems the absorption of an incoming photon creates a local hot spot, which eventually leads to the breakdown of superconductivity in the entire sample^44^. The transition into the resistive state is usually accompanied by the appearance of highly mobile vortex-antivortex pairs. Here, we investigate if such a transient dynamic state can also be created with the aid of a structured THz field pulse. The external current is introduced into our system by solving the continuity equation

10 $$\begin{aligned} 0 = \nabla \cdot {\mathbf {j}}_\mathrm {s} - \sigma \Delta \phi \end{aligned}$$

with $\sigma = 38\,(\mu \Omega \mathrm {m})^{-1}$ and boundary conditions

11 $$\begin{aligned}&\Psi = 0, ~~ \text {on} ~~ \partial \Omega _e \end{aligned}$$

12 $$\begin{aligned}&- \sigma \nabla \phi \cdot {\mathbf {N}}_e = j_e, ~~ \text {on} ~~ \partial \Omega _e \end{aligned}$$

where $\Omega _\mathrm {v}$ and $\Omega _\mathrm {e}$ are the SC/vacuum and SC/electrode boundaries, and ${\mathbf {N}}_\mathrm {v}$ and $\mathbf{N}_\mathrm {e}$ are the corresponding surface normals^45^. In our case the external current flows along the *y*-direction and the superconductor/electrode boundaries are located at $y=\pm \,250\,$nm. In a first step the external bias is quasi-adiabatically increased until the critical current $j_\mathrm {c}$ is found. In our case the system is observed to transit into the resistive state for $j_\mathrm {e}=j_\mathrm {c} = 0.45\,$GA$/\mathrm {cm}^2$. Next, the external current is fixed to $j_\mathrm {e}=0.95j_\mathrm {c}$ and the system is allowed to relax into the equilibrium fully. Finally, the current-driven superconductor is subjected to the STE emitted field pulse with a frequency $f=10\,$THz and a ramping factor $a_T = 0.166$Tμm/ps. In the simulations, the value of the transport current is chosen relatively high, which we expect to render the superconductor more susceptible to the applied THz radiation. In a real experimental setup, the applied radiation field may heat the sample locally, providing an additional driving factor for the breakdown of superconductivity, an effect which is not taken into account here.

Fig. 9 shows the general trend of the superconducting dynamics in a square sample with a side length $a=250\,$ nm under the application of the STE generated field pulse with $f=10\,$THz and $a_T = 0.166$Tμm/ps. The field is ramped up slower than in the previous calculations and the order parameter is only gradually affected at first. Once the field amplitude is high enough, the system transits quickly into the normal conducting state. The transition by itself was not observed to be accompanied by the emergence of vortex-antivortex pairs, as in the case of the classic single-photon detector. Instead, the breakdown of superconductivity starts at the superconductor/electrode boundaries, where the order parameter is already suppressed. The magnetic field of the THz field pulse drives the growth of the normal conducting domains until the entire material becomes normal conducting again. This behavior can also be observed for THz field pulses of lower frequency and ramping rate (not shown here). An important factor is the relatively small size of the system. In larger superconductors, the THz field might affect the order parameter evolution differently and vortex formation might become possible. This might be especially interesting if we take the local heating of the sample into account. In this way, the combination of a transverse current flow and a quench of the locally induced hot spot could lead to the formation of vortex avalanches which are characteristics for the applied THz field pulse^46^.

## Conclusions

In summary, spintronic THz emitters deliver fields that are spatially and vectorially textured on the nanometer scale and are useful for studying the linear and nonlinear subgap response of superconductors to phase-locked transverse and longitudinal fields, which is particularly interesting for superconductors with collective dynamics in the THz range such as iron pnictides^47^, cuprates^48,49^, MgB_2_ or NbSe_2_^48,50^. Here, we showed how the superconducting density and the superconducting current in nanometer scale samples with different geometries could be driven spatio-temporally. Also, the vortex lattice dynamics, as well as the transport properties, can be influenced by the structured THz field pulses. Further aspects for future/ongoing studies concern the possibility of local heating and also the role of proximity effects when the STEs are in close vicinity of the superconducting sample such that the stray fields of the ferromagnet couple to the superconducting dynamics and the STEs are effectively coupled to the superconducting dynamics.

## Methods

**Figure 10:**
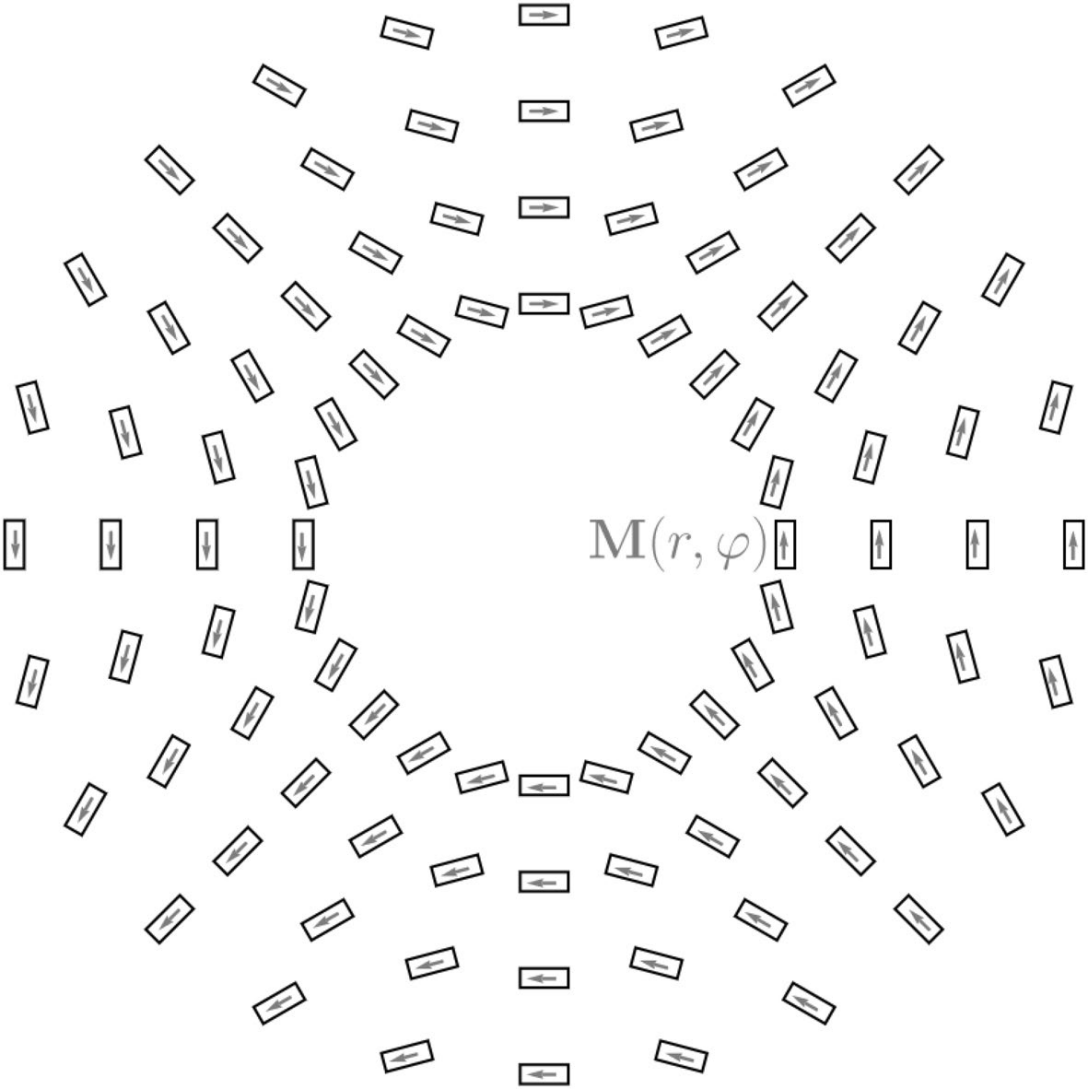
Top view of the magnetization direction (arrows) (${\mathbf {M}}(r,\varphi )$) of the individual STEs, which is stabilized by intrinsic magnetic interaction (such as the shape anisotropy). The topology of the emitted THz fields at the center of the texture remains basically preserved (but with accordingly enhanced intensity) when the basic central unit (shown also in Fig. 1) is periodically repeated with a larger radius as to enclose the micrometer-sized metastructure.

The fields used above are generated by the assembly of spintronic terahertz emitters (STEs) in a fashion that inherits a twist in the magnetization on a circular path, as shown in Fig. 10. Increasing the radius of this path without changing the topology results in the metastructure shown by Fig. 10. The THz field amplitude is enhanced but the topology of the THz field is preserved and characterized by topological charge $q=\frac{1}{2\pi }\int _0^{2\pi }\left[ \partial _{\varphi }{\mathbf {m}}\times {\mathbf {m}} \right] _z \,\mathrm {d}\varphi$ (with ${\mathbf {m}}={\mathbf {M}}/M$). The individual STE is a trilayer stack consisting of $2\,$nm W, $1.8\,$nm CoFeB, and $2\,$nm Pt. These emitters were examined in Ref.^18^ and are the standard STEs used in experiments. They give rise to a broadband pulse ranging from 0 to 30 THz propagating along the z-axis. In conventional experimental setups, magnetic fields stabilize the magnetization of large STEs along a particular axis. In contrast, we use the shape anisotropy of the emitters by shrinking them in size to a rectangular shape (see Fig. 1a). Increasing the number of emitters on the circular path, we take the limit of a space-filling annulus placed on a sapphire substrate. For simulation purposes, we exploit the fact that the rotation of the magnetization along the annulus can be calculated as a superposition of two setups with the azimuthal mode numbers $m=2$ and $m=-2$. The generated electric field polarization is perpendicular to the magnetization of the CoFeB layer and the growth direction of the trilayer stack. The emitted field at the surface of the annulus can therefore be described by

13 $$\begin{aligned} \mathbf { {E}}_0=&\, E_0\left[ \frac{1}{2}\left( \,\hat{\varvec{\mathrm {e}}}_{\rho } + \mathrm {i}\,\hat{\varvec{\mathrm {e}}}_{\varphi }\right) \mathrm {e}^{\mathrm {i}m \varphi } + \frac{1}{2}\left( \,\hat{\varvec{\mathrm {e}}}_{\rho } - \mathrm {i}\,\hat{\varvec{\mathrm {e}}}_{\varphi }\right) \mathrm {e}^{-\mathrm {i}m \varphi }\right] \nonumber \\ =&\, E_0\left[ \cos (m\varphi ) \,\hat{\varvec{\mathrm {e}}}_{\rho } - \sin (m\varphi ) \,\hat{\varvec{\mathrm {e}}}_{\varphi }\right] \nonumber \\ =&\, E_0\left[ \cos (\{m-1\}\varphi )\,\hat{\varvec{\mathrm {e}}}_x - \sin (\{m-1\}\varphi ) \,\hat{\varvec{\mathrm {e}}}_y\right] \,. \end{aligned}$$

Here, $E_0$ is the amplitude of the electric field, $m-1$ is equal to the topological charge *q*, and $\,\hat{\varvec{\mathrm {e}}}_{\alpha }$ are the unit vectors along $\alpha$.

## Electromagnetic simulations

For the simulation of the Maxwell equations, the following conditions were assumed: The cylindrical system measures $60\,\upmu$m in height and a radius of $50\,\upmu$m, while the thickness of the substrate is $10\,\upmu$m and that of the sample is $5.8\,$nm. The distance from the emitters to the center of the metastructure is $25\,\upmu$m, and the relative permittivity of sapphire is known to be $\varepsilon _{\mathrm {r,Sap}}=11.7$. For the combined multistack, one finds a conductivity of $\sigma =\tfrac{\sigma _0 \Gamma ^2}{\omega ^2+\Gamma ^2}$ and a relative permittivity of $\varepsilon _{\mathrm {r,M}}=-\Gamma \tfrac{\sigma _0}{\varepsilon _0 (\omega ^2+\Gamma ^2)}$ (see Ref.^51^) with $\Gamma = 2\pi \cdot 80\,$THz and $\sigma _{0} = 2.1\frac{{{\text{MS}}}}{{\text{m}}}$. Concerning the resolution of the FEM calculation, the minimal element size is $2.5\,$nm, while the maximal element size is $1.5\,\upmu$m. At the outer regions, a second order scattering boundary condition was used. The calculations were performed in the frequency domain for a range of frequencies $f\in \left[ 1, 50\right] \,$THz, employing a step size of $1\,$THz. With the individual STE width $w_{\text {STE}} = 1.25\,\upmu$m and length $l_{\text {STE}} = 5\,\upmu$m, the width of the annulus is given as the arithmetic mean value $\frac{w_{\text {STE}} + l_{\text {STE}}}{2}$. The simulations were done using the RF module of COMSOL® ^52^.

## Simulation of the time-dependent Ginzburg–Landau equation

The field delivered by the electromagnetic simulations at $f=\omega /2\pi =10\,$THz is fitted to the form ${\mathbf {A}}_T = A_{T0} {\mathbf {A}}_{T1}(\rho ,\varphi ,z,t)$ with

14 $$\begin{aligned} {\mathbf {A}}_{T1} =&\, \left[ A_{\rho 1}\cos (\omega t) + A_{\rho 2}\sin (\omega t) \right] \cos (2\varphi ){\mathbf {e}}_{\rho } \nonumber \\&\,+ \left[ A_{\varphi 1}\cos (\omega t) + A_{\varphi 2}\sin (\omega t) \right] \sin (2\varphi ){\mathbf {e}}_{\varphi } \nonumber \\&\,+ \left[ A_{z1}\cos (\omega t) + A_{z2}\sin (\omega t) \right] \cos (2\varphi ){\mathbf {e}}_{z}. \end{aligned}$$

Based on the numerical simulations of the STE generated THz fields the space and the time dependent functions $A_{\rho i}$, $A_{\varphi i}$ and $A_{z i}$ are obtained from a polynomial fit of 8th order. The prefactor $A_{T0}$ is a time-dependent linear function. Eqs. (1) and (2) were simultaneously solved by using a semi-implicit Galerkin-mixed finite element method (FEM)^40^ and the FEM-software Fenics^53^. The stability of the methods have been tested for various setting^54,55^.

### Fourier analysis of the pulse

To gain insight in the frequency range and the dominant frequency contributions when ramping the THz field, we inspect the Fourier transform of the ramped vector potential. The full form of the time dependencies is

15 $$\begin{aligned} {\mathbf {A}}_T({\mathbf {r}},t)&= f(t) \left[ \Re {\mathbf {A}}_T({\mathbf {r}}) \cos (\omega _0 t) - \Im {\mathbf {A}}_T({\mathbf {r}}) \sin (\omega _0 t) \right] \end{aligned}$$

16 $$\begin{aligned} \text {with } f(t)&= {\left\{ \begin{array}{ll} 0 &{} t\le 0\\ a_T t &{} 0<t\le A_{T0} / a_T\\ A_{T0} &{} t> A_{T0} / a_T \end{array}\right. }. \end{aligned}$$

Therefore, the Fourier transform for the cosine term (the sine term can be calculated analogue) has the form

17 $$\begin{aligned} {\mathcal {F}}[f(t)\cos (\omega _0 t)] = \frac{a_T \left( \frac{\pi \left( \omega -\omega _0\right) ^2 A_{\text {T0}} \delta \left( \omega -\omega _0\right) }{a_T}+\mathrm {e}^{\frac{\mathrm {i}\left( \omega -\omega _0\right) A_{\text {T0}}}{a_T}}-1\right) }{2 \sqrt{2 \pi } \left( \omega -\omega _0\right) ^2} + \omega _0\rightarrow -\omega _0. \end{aligned}$$

This shows, that the spectrum of the used time function has delta peaks at the frequency $\omega _0=2\pi \cdot 10\,$THz and a $\vert {\mathcal {F}}[f(t)\cos (\omega _0 t)]\vert \le \frac{a_T}{\sqrt{2\pi }(\omega -\omega _0)^2}$ decay close to it ($\omega \ne \omega _0$).

## Data Availability

The data for this study are available from the authors upon request.

## Code availability

Details of the codes used to generate the THz fields and to solve for the time-dependent Landau–Ginzburg equations are available from the authors upon reasonable request.
